# Proceedings of the inaugural Dark Genome Symposium: November 2022

**DOI:** 10.1186/s13100-023-00306-5

**Published:** 2023-11-21

**Authors:** Jef D. Boeke, Kathleen H. Burns, Katherine B. Chiappinelli, Marie Classon, John M. Coffin, Daniel D. DeCarvalho, Joseph D. Dukes, Benjamin Greenbaum, George Kassiotis, Sarah K. Knutson, Arnold J. Levine, Avindra Nath, Sophie Papa, Daniel Rios, John Sedivy, David T. Ting

**Affiliations:** 1 Institute for Systems Genetics, NYU Langone Health, New York, NY 10016 USA; 2 Department of Biomedical Engineering, NYU Tandon School of Engineering, Brooklyn, NY 11201 USA; 3 Department of Biochemistry and Molecular Pharmacology, NYU Langone Health, New York, NY 10016 USA; 4 Department of Pathology, Dana-Farber Cancer Institute, Harvard Medical School, Boston, MA USA; 5 Department of Microbiology, Immunology and Tropical Medicine, The George Washington University, Washington, DC USA; 6 Pfizer Centre for Therapeutic Innovation, San Diego, USA; 7 Department of Molecular Biology and Microbiology, Tufts University, Boston, MA 02111 USA; 8 Princess Margaret Cancer Centre, University Health Network, Toronto, ON Canada; 9 Department of Medical Biophysics, University of Toronto, Toronto, ON Canada; 10 Enara Bio Limited, Magdalen Centre, 1 Robert Robinson Avenue, The Oxford Science Park, Oxford, OX4 4GA UK; 11 Computational Oncology, Department of Epidemiology and Biostatistics, Memorial Sloan Kettering Cancer Center, New York, NY 10065 USA; 12 Retroviral Immunology Laboratory, The Francis Crick Institute, London, UK; 13 Department of Infectious Disease, Faculty of Medicine, Imperial College London, London, UK; 14 Rome Therapeutics, 201 Brookline Avenue, Suite 1001, Boston, MA USA; 15 Simons Center for Systems Biology, Institute for Advanced Study, Princeton, NJ USA; 16 Section for Infections of the Nervous System, National Institute of Neurological Disorders and Stroke, National Institutes of Health, Bethesda, MD USA; 17 School of Cancer and Pharmaceutical Sciences, Faculty of Life Sciences and Medicine, King’s College London, London, UK; 18 Center on the Biology of Aging, Brown University, Providence, RI USA; 19 Department of Molecular Biology, Cell Biology and Biochemistry, Brown University, Providence, RI USA; 20 Department of Medical Oncology, Massachusetts General Hospital, Harvard Medical School, Boston, MA USA

## Abstract

In November 2022 the first Dark Genome Symposium was held in Boston, USA. The meeting was hosted by Rome Therapeutics and Enara Bio, two biotechnology companies working on translating our growing understanding of this vast genetic landscape into therapies for human disease. The spirit and ambition of the meeting was one of shared knowledge, looking to strengthen the network of researchers engaged in the field. The meeting opened with a welcome from Rosana Kapeller and Kevin Pojasek followed by a first session of field defining talks from key academics in the space. A series of panels, bringing together academia and industry views, were then convened covering a wide range of pertinent topics. Finally, Richard Young and David Ting gave their views on the future direction and promise for patient impact inherent in the growing understanding of the Dark Genome.

In the following proceedings paper the contributing speakers, panel introduction speakers and panel moderators have summarized the topics covered.

Jef Boeke: *The Dark Matter Project: writing mammalian DNA*

The dark genome is littered with the dead bodies of retrotransposons – and although they may have lost their ability to jump around the genome, those insertions might have consequences for phenotype. However, retrotransposon insertions in mammalian introns are widely assumed to be of no consequence, since introns simply get spliced out. Mammalian retrotransposons include Long interspersed nuclear elements (LINEs) which include the machinery for retrotransposition and encode the machinery for their movement to new sites – they are thus called “Autonomous”. Nonautonomous human elements that exploit the LINE-encoded machinery include the abundant Arthrobacter luteus (*Alu)* restriction endonuclease sequence, and the less abundant (ca. 1000 copies) SINE-VNTR-*Alu*s (SVA) sequence. However, we have made two sets of findings that challenge this widely held assumption. One of these relates to the human *Alu* Sequence, an element found in over a million copies in the human genome; many of those copies are intronic. Could any of these intronic copies have specific effects on gene expression? Bo Xia in the Yanai and Boeke groups explored the loss of the tail in hominids, one of the main anatomical evolutionary changes to have occurred along the lineage leading to humans. Morphological reprogramming in ancestral hominoids has been long considered to have accommodated a characteristic style of locomotion and contributed to the evolution of bipedalism in humans. However, the precise genetic mechanism that facilitated tail-loss evolution in hominoids remains largely unknown. Primate genome sequencing projects have made possible the identification of causal links between genotypic and phenotypic changes, and could enable the search for hominoid-specific genetic elements controlling tail development. Bo found evidence that tail-loss evolution was mediated by the insertion of an individual *Alu* element into the genome of the hominoid ancestor. He demonstrated that this *Alu* element – inserted deep in the middle of an intron of the T-Box Transcription Factor T *(TBXT)* gene (also called *T* or *Brachyury*) – pairs with a neighboring *Alu* element encoded in the reverse genomic orientation in the downstream intron and leads to a hominoid-specific alternative splicing event. Follow up studies in the mouse suggest that alternative splicing variation such as those caused by the *Alu* insertion can influence the length of the tail. This provides strong presumptive evidence that this particular *AluY* insertion was a pivotal molecular development of loss of the tail in hominoids, as it is only observed in the hominoid lineage and not in other (tailed) primates [1].

A second case relates to the rare human disease X-linked dystonia parkinsonism (XDP), which affects males from the island of Panay in the Philippines in their fourth decade of life. This devastating disorder, which is uniformly fatal and has no cure or even treatment, is caused by an ancestral SVA retrotransposon insertion deep in the middle of a large intron (intron 32) of the essential TATA-box binding protein associated factor 1 (*TAF1)* gene. This SVA insertion presumably occurred once in a single female from Panay (a so called “founder effect”) within relatively recent history, and the subsequent inheritance of the insertion by male offspring has revealed untold suffering in those unlucky enough to inherit it. SVA is a human-specific nonautonomous retrotransposon which is not very well studied. In order to better understand the disorder and its molecular etiology, and potentially to test therapeutics for treating it, a practical animal model would be useful. We used “Big DNA” technology, developed in our lab, to solve a thorny problem – how do you engineer the gene to contain a potentially toxic retrotransposon insertion in an essential gene on the X chromosome in mouse embryonic stem cells which are male. We developed a strategy for doing this using a conditional insertion carrying a “convertible allele” controlled by a recombinase. Mice derived from this “convertible allele” by breeding them with driver mice that convert the allele to partially human, in a tissue specific manner and observed phenotypes in the appropriate male mice (only) that resemble certain human XDP symptoms. Thus, a system now exists to better dissect the impact of a disease causing SVA insertion, in the appropriate tissues of a living animal.

Kathleen H. Burns: *Transposable elements in cancer*

This seminar began with an overview of the contributions of transposable elements and interspersed repeat sequences to human genome composition. The ongoing activity of LINE-1 sequences was highlighted, particularly with respect to its contributions to genetic diversity in human populations via retrotransposition of LINE-1, *Alu* and SVA sequences. Rarely, but recurrently, mobile element insertions generate loss-of-function alleles causing genetic disease, such as the hemophilia allele described by Kazazian and colleagues in 1988 [2]. More recent work, by our lab and others, has shown that commonly occurring insertion polymorphisms can also impact heritable disease risk by altering mRNA splicing or gene expression [3–5].

The primary focus of our discussion then turned to LINE-1 dysregulation in human cancers. Across a wide variety of commonly occurring, p53-mutated epithelial cancers, LINE-1 open reading frame 1 protein (ORF1p) can be detected in tissue biopsies [6]. Moreover, we shared very recent evidence that this candidate ‘binary’ cancer biomarker can be appreciated in the peripheral blood of patients with advanced cancers using ultrasensitive protein detection assays [7]. These findings raise the possibility that LINE-1 expression may be leveraged for early detection or disease monitoring. We next reviewed data that LINE-1 expression is associated with somatic retrotransposition of LINE-1 sequences in cancers: several independent groups have now converged on the recognition that LINE-1 ORF2p is a mutator of cancer genomes [8]. We described published and unpublished data from our group that LINE-1 expression may incite chromosomal instability (CIN) and shape selective pressures on cells within cancers and premalignant lesions. For example, promoting tumor protein p53 (p53) loss or imposing requirements for DNA repair [9]. The paradigm that epigenetic dysregulation of LINE-1 is a hallmark of cancers where its expression promotes a ‘mutator phenotype’ and imposes molecular dependencies raises therapeutic implications, presenting important future directions for our field.

Arnold J. Levine: *Review of the role of Tp53 gene and protein in regulating epigenetic changes and LINE-1 gene expression*

The first demonstration that the p53 protein plays a role in the regulation of epigenetic changes came from the Jaenisch laboratory [10]. They created mice with a conditional cyclization recombinase (CRE) mediated deletion of the DNA methyltransferase 1 (DNMT1) gene (the copy DNA methy-cytosine transferase). This enzyme adds a methyl group (CH3) onto cytosines in CpG dinucleotides opposite GpC-methyl cytosine on the paired DNA strand after DNA replication, duplicating the epigenetic marks. In response to eliminating both DNMT1 genes with Cre cells were shown to replicate twice producing cells with no methylation of the C-residues in CpG dinucleotides and those cells died of apoptosis. When Tp53 genes were deleted from these DNMT1 deleted genes the cells failed to undergo apoptosis divided and ultimately became transformed and tumorigenic. These experiments suggested that alterations in epigenetic marks on the DNA were detected by p53 and these cells were killed by a p53 mediated apoptosis. This interpretation is confirmed by the results of the Yamanaka experiment [11]. In this case four different transcription factors are added to a fibroblastic cell in culture and through cell division the epigenetic marks are erased, and the cells dedifferentiate into a stem cell that can be reprogramed to produce many different cell and tissue types. When this occurs in cell culture the efficiency of stem cell production is very low (0.1-1.0%) and the time taken to produce these stem cells in culture is commonly one week to one month. However, in the absence of the wild type p53 protein (using a temperature sensitive p53 protein) the efficiency increases up to 80% and the time to produce stem cells decreases to days. With a temperature sensitive p53 protein stem cells are produced at high frequencies in short times only at the non-permissive temperature. Temperature shifts map the times of cell death by apoptosis [12]. A third type of experiment also supports these ideas. The drugs azacytidine or decitabine (the deoxyribose form of the drug) are faulty incorporators into RNA or DNA at cytidine resides and prevent methylation of cytosines at CpG dinucleotides. These drugs will kill cells more efficiently that have lost the p53 wild type functions sparing cells that have wild type p53 functions [13]. A similar observation has been made in tumor xenografts in mice and in humans [14]. The reciprocal relationship between mutant p53 and epigenetic changes seems to be operative as well [15]. When p53 mutations occur in both alleles of the Tp53 gene epigenetic changes occur in selected regions of the genome and this results in Line-1 ORF-1 and 2 transcriptional gene expression. Presumably when LINE-1 transcription occurs in a cell with wild type p53 protein the cell will be eliminated by apoptosis or other methods of cell death mediated at least in part by the p53 pathway. As this happens over a lifetime, antibodies are produced by the adaptive immune system, (or perhaps the innate immune system) that give many individuals a background low level of antibodies directed against ORF-1 whose levels can increase as a cancer develops (talk by the Gudkov group). It is not uncommon to detect increasing levels of Tp53 mutations in people as they age [16].

The exact mechanism(s) by which the p53 protein or pathway senses changes in epigenetic marks is not yet clear.

John Coffin: *Endogenous Retroviruses are not Retrotransposons: The Case of HERV-K (HML-2)*

The genomes of all vertebrates – and many invertebrates – are laced with endogenous retroviruses (ERVs), segments of DNA proviruses derived from retroviral infection of the germline of a distant ancestor. In humans, such endogenous retroviruses (HERVs), numbering about 80,000 distinct elements, are all defective, likely the consequence of evolutionary selection against individuals with intact proviruses, those capable of giving rise to pathogenic infectious virus during development. While it is commonplace to consider ERVs as “retrotransposons,” implying intracellular propagation within the germ line, I consider this to be a highly misleading classification, because of the large differences in biology and evolution between ERVs and true retrotransposons (such as LINEs). ERVs have several features that render them incompatible with an intracellular replication cycle, such as delaying proteolytic processing of virion proteins until after budding and release of virions. Thus, while retrotransposons make a home in the genome, for ERVs, the genome is better considered as a graveyard where the losers are buried, providing us with a fossil record of long-extinct pandemic infection of our distant ancestors. Nevertheless, A few of the germline proviruses have been coopted for functions useful to the host, such as syncytins, ERV envelope (*env)* genes that play an important role in early fetal development. Perhaps the most common (and most underappreciated) ERV function is as a restriction factor to prevent infection with a related exogenous virus, for example by receptor blockade due to expression of an Env protein.

Our laboratory has been studying the most recently active HERV, human endogenous retrovirus-K (HERV-K/HML-2), which appears to have gone extinct in the human lineage within the last million years (although much more recently in the gorilla lineage), leaving about a thousand proviruses in our genome. 900 of these proviruses have been reduced to solo LTRs, and the remaining 100 or so are all defective for replication, although some are still capable of expressing one or a few viral gene products, and even non-infectious virus-like particles (VLPs). Much of the recent literature on HML-2 expression is based on two implicit assumptions: (1) That any detected HML-2 expression implies the presence of all functional gene products; (2) That expression is confined to disease states, particularly cancer. Neither of these is correct. Of all the proviruses with 2 long terminal repeats (LTRs), no two are alike in the pattern of inactivating mutations, and the potential for functional protein expression varies considerably from one to the next. Recently, we have used the Genotype-Tissue Expression (GTEx) RNA-seq database of 54 non-diseased tissues from 900 donors to assess the expression of all known HML-2 proviruses [17]. We found expression of one or more proviruses in one or more tissues, no two of which showed the same pattern. Of particular interest is the 8 + million-year-old provirus on chromosome 3 at 3q12.3, which was expressed in all 53 solid tissues tested due to a post-integration mutation in the 5’LTR. This mutation created a binding site for a common transcription factor, allowing widespread expression of a variant gag protein. We are now pursuing the hypothesis that this protein was coopted as a factor to restrict replication of the now extinct HML-2 virus.

George Kassiotis: *Antibodies to endogenous retroviruses underpin lung cancer immunotherapy*

The effectiveness of immunotherapies in a sizeable proportion of patients with several cancer types has not only transformed patients’ lives, it has also validated a long-held belief that the immune system can be stimulated to protect against tumors. While most of this protective effect has been traditionally attributed to T cells, a growing body of evidence points to a possible contribution of B cells and, by extension, antibodies to immune protection against tumors and to the success of immunotherapies. However, the mechanism by which B cells may mediate their effects is still incompletely understood. We hypothesized that this effect is attributable to the production of anti-tumor antibodies and set out to identify their putative targets.

The team used a mouse model for lung adenocarcinoma, newly generated by Julian Downward’s group at the Francis Crick Institute, that, unlike earlier models, is highly immunogenic, and presented evidence for the formation of tertiary lymphoid structures around lung tumors and for local germinal center reactions [18]. These were accompanied by the induction of highly protective anti-tumor antibodies. Perhaps unsurprisingly for a mouse cancer model, the team demonstrated that anti-tumor antibodies spontaneously induced during the course of tumor challenge targeted the envelope glycoproteins of infectious murine leukaemia viruses (MLV) “resurrected” from defective endogenous retrovirus (ERV) precursors in the tumor cells. Expression of MLV envelope glycoproteins was both necessary and sufficient for the immunogenicity of lung cancer cells and the induction of anti-tumor antibodies. Moreover, these antibodies were significantly boosted following checkpoint blockade, which further enhanced their protective capacity, suggesting that immunotherapies work at least in part through enhancement of the anti-tumor antibody response.

Given the evolutionary divergence of murine and human ERVs, the team then explored if phylogenetically unrelated ERVs in humans could serve the same purpose as the murine ones. By probing ERVs with an intact or near intact envelope gene, they identified three distinct proviruses with high expression in lung cancer samples, one of which, a member of the HERV-K(HML-2) family, additionally showed specific upregulation in lung adenocarcinoma (LUAD). Working with Charles Swanton’s group at the Francis Crick Institute and using bespoke assays for antibodies against these envelope glycoproteins, the team reported spontaneous antibody responses specifically in LUAD and specifically to HERV-K(HML-2). The team further reported that antibodies to HERV-K(HML-2) envelope glycoproteins were induced or further boosted, if they pre-existed, in the weeks following immunotherapy of LUAD patients. Finally, in collaboration with colleagues at the Samsung Medical Centre in Seoul, Republic of Korea, they found that pre-treatment expression of the particular HERV-K(HML-2) provirus on Chromosome 1q22 predicted the outcome of immunotherapy in a larger cohort of LUAD patients. The reported findings [18] raised the possibility that the reactivation of normally repressed ERVs provides non-mutated cancer-associated targets of viral origin for immune recognition of transformed cells, with implications for improvement of cancer immunotherapies.

Avindra Nath: *Role of retroviruses in Amyotrophic Lateral Sclerosis*

The possibility that retroviruses may be implicated in the pathophysiology of motor neuron disease or amyotrophic lateral sclerosis (ALS) dates to the 1960’s, before the term “retrovirus” had been coined. PG Stansly described a “paralytic disease involving upper and lower motor neurons associated with virus-induced neoplasms of the mouse” [19]. He elaborated the history of the discovery, the clinical features, the pathology, the long latency, and the effect of the age of the host on the development of the syndrome by a filterable agent that was also associated with lymphomas. Subsequently, Murray Gardner and colleagues isolated the infectious agent and identified it as MLV. The pathology and clinical course resembles that of ALS with a long latency period and absence of inflammation [20]. Extensive work followed describing the molecular pathophysiology of MLV associated motor neuron disease [21–23]. This also initiated the hunt for retroviruses in patients with ALS.

Several research groups have consistently demonstrated the presence of reverse transcriptase activity in patients with ALS [24–27]. Further it has been shown that HIV and HTLV-I infection can also cause an ALS-like syndrome in some individuals [28, 29]. However, attempts to isolate a retrovirus from ALS patients have consistently failed. We and others have shown that an endogenous retrovirus HERV-K (subtype HML-2) gets activated in the brain and spinal cord of a subgroup of patients with ALS [30, 31]. Further, the loci activated in ALS have open reading frames for the viral proteins which include the envelope, reverse transcriptase and gag proteins [32]. Some investigators have not been able to demonstrate any difference between ALS and controls for HERV-K activation. We found that the envelope protein is toxic to neurons and transgenic animals that express the protein in neurons develop an ALS like syndrome [30]. The mechanism of neurotoxicity involves the cleavage of the signal peptide with nucleolar localization and disruption of protein synthesis (unpublished). Envelope protein can also be identified in the cerebrospinal fluid (CSF) of ALS patients [33] who develop an immune response against the virus, and analysis of the antibody binding epitopes shows that over the course of the illness there is evidence for epitope spreading with unique antibodies directed against the transmembrane domain of the protein in ALS patients only [34]. Through the process of drug screening, others and we have identified several antiretroviral drugs with variable efficacy against HML-2 [35, 36]. Of these a reverse transcriptase inhibitor, Abacavir has the best efficacy [37]. An open label study using Triumeq (abacavir, dolutegravir, and lamuvidine) in ALS patients showed that over a period of 24 weeks, the HML-2 viral load decreased in a subset of patients who also showed slower progression of the disease [38] (Garcia-Montojo et al., 2021). Based on these results, a double blind, placebo-controlled study has been initiated in Europe, Australia and New Zealand. However, there is a great need for better and more specific antiretrovirals against this virus.

John Sedivy: *Retrotransposon Activation and Consequences in Senescent Cells and Aging Tissue*

Aging is associated with extensive remodeling of the epigenome [39]. The age-related changes are complex but show a consistent trend for loss of constitutive heterochromatin [40–42]. Cellular senescence is an arrest of proliferation, studied primarily in mammals, which can be elicited by a variety of stresses including DNA damage [43]. While it has beneficial functions (such as tumor suppression), senescent cells accumulate in most tissues with age, and are an important component of the overall aging process [44]. In particular, due to their profound proinflammatory phenotype [45], senescent cells have been causally linked with many age-related pathologies and diseases [46]. Senescence is accompanied by widespread opening of heterochromatic regions, some of which were mapped to retrotransposable elements and associated with their derepression [47].

Retrotransposition in the soma appears to be very low. Line-1 mRNA is found in the adult brain [48–50] and neural stem cells can support retrotransposition [51], but insertions in adult neurons are only in the range of 0.5-1.0 events per cell [52–55]. In other tissues retrotransposition appears to be significantly lower [55]. Cancer is associated with increased LINE-1 expression [56] and many insertions, in some cases hundreds in an individual tumor, have been reported [8, 57–59]. Most appear to be passenger events in non-coding regions [60, 61].

In eukaryotic cells nucleic acid species such as DNA or double stranded RNA (dsRNA) in the cytoplasm are perceived as invading pathogens. In vertebrates this triggers the Type I Interferon (IFN-I) response: expression of interferons alpha and beta to alarm neighboring cells, a variety of cytokines and chemokines to communicate with the immune system, a large number of factors that interfere with viral replication, and in some cases cause cell death [62]. Cytoplasmic LINE-1 DNA is present in senescent cells and in tissues of mice in association with normal aging [63, 64]. Importantly, LINE-1 DNA was identified in association with the DNA sensor cyclic GMP-AMP Synthase (cGAS) [64].

Treatment with nucleoside reverse transcriptase inhibitor (NRTI) drugs, or a knockdown of LINE-1 mRNA with shRNAs against active LINE-1s, decreased cytoplasmic LINE-1 DNAs and ameliorated cGAS and IFN-I activation [63–65], indicating that cytoplasmic LINE-1 cDNA can be a potent trigger of an IFN-I response. The mechanisms by which cytoplasmic LINE-1 cytoplasmic DNAs are produced are not yet understood. Normally LINE-1 reverse transcription occurs in the nucleus by target-primed reverse transcription (TPRT) [9, 66]. One possibility is that cytoplasmic DNA species are abortive TPRT products that are somehow exported from the nucleus. Alternatively, reverse transcription could occur directly in the cytoplasm. Both purified ORF2 and LINE-1 RNPs purified from cells can reverse transcribe in vitro if provided with a primer [67, 68].

One consistent feature has been the efficacy of NRTIs, in multiple models and situations, to alleviate the effects of retrotransposable element activation. This finding is intriguing given that some aspects of the retrotransposable elements lifecycle, such as transcription of the elements, or stimulation of RNA sensors, should not be directly affected by NRTIs. One explanation might be downstream cross-talk between RNA and DNA sensing pathways and the existence of overall self-tolerance thresholds. Much more research needs to be done in this area, and it will thus be important to determine, for any given disease, which family of retrotransposable elements is being activated and whether DNA or RNA sensing pathways are engaged.

Panels.

Moderator: Joe Dukes

*Mapping the dark genome*

Opening speaker and panellist: Ben Greenbaum

Panellists: Rachel O’Neill

Menachem Fromer

Alice Lee

Samuel Lukowski

The first panel discussion explored the history and challenges of mapping traditionally thought to be non-coding regions of the human genome, where successes in this space emerged from and some of the remaining challenges especially in moving towards applications.

Benjamin Greenbaum provided an introductive overview talk on the topic. The main questions raised were what is the structure of information in the dark genome, what aspects continue to evolve and how do we assign function to repeats in healthy tissue, in disease and under therapy? In the pre-next generation sequencing era repeats were assessed by the rates of DNA reannealing. However next generation sequencing has opened an era of precise genome mapping and comparative genomics [69]. It has become clear that long-read sequencing has uncovered missing dark genome events, and likewise that biased transcriptomics have missed repeats in cancer with immune consequences [70, 71].

Cancer was highlighted as an area where the underassaying of the repeatome has led to a loss of detection of consequential activity. Repeats which are expressed in cancer are being missed [70], and total RNAseq is needed to quantify them [71]. Recent work has shown that LINE-1 continues to reverse transcribe in human cancers [8, 72], particularly those associated with p53 mutation loss of function [73], indicating events that are missed due to lack of whole genome sequencing. Likewise, HERV-K expression has been associated with immunotherapy response in renal cell [74] and bladder cancer [71]. Immunostimulatory repeats which engage in viral mimicry by displaying viral associated molecular patterns are also being under quantified [71]. Emerging methods from statistical physics are likely able to predict their immune function when sequenced [75].

Rachel O’Neill kicked off the discussion by reminding the audience that despite what was dubbed the publication of the entire human genome in 2001 [76, 77], it was missing around 8% of the sequence due to limitations in the sequencing methods and technologies. Only in 2022, through the impressive collaborative efforts of the Telomer-to-Telomer (T2T) consortium along with technological advances, were this team able to finally publish hg38, a fully mapped human genome [78]. Some of the key challenges to overcome were the repetitive elements as well as sequencing through centromeres. A reoccurring theme of the panel discussion was how limited short read sequencing is for mapping and assembling genetic sequences. The advent of long read sequencing, especially through Oxford Nanopore and PacBio’s platforms, have been key technological breakthroughs that have helped to unlock the ability to map such sequences. In addition, improvements in computational tools have been critical to success. The T2T group, consisting of collaborative efforts from around 100 different labs - in the absence of direct funding for this work, was touted as the poster child for how the field needs to continue to operate openly to drive efficient success.

Some of the key challenges that remain in the field were noted to be around the annotation of sequences and associated pipelines, composite repeats and variation in human repeat elements among individuals. The next frontier must also interrogate the evolutionary landscape to understand, especially in genomic hotspots of the repeateome, our evolutionary history to better elucidate function and ultimately application.

Alice Lee brought these points towards a discussion on population scale data, reiterating that long read sequencing of repeats is a necessary means to adequately generate this data. In particular, rare but important transposons, such as those only present in a small proportion of cells, need to be mapped and further characterized in order to better understand human health and disease. The challenge of annotation was again highlighted, here in the context of transposon events. The other challenge in looking at rarer events and especially solving this with single cell analyses, is the ability to confidently distinguish between true events rather than artefacts of working with limited depth. Samuel Lukowski picked up on this point highlighting that more whole transcriptome sequencing needs to be performed and, with approaches such as long read RNA-seq at the bulk RNA and/or single cell level, would greatly enhance our view of the tissue-, cell type-, and disease-relevant events.

Menachem Fromer turned the discussion to the challenge of mapping sequence data using LINE1 as an example. Here a major challenge is how to accurately map where in the genome a sequence is coming from and what the mechanisms of control are, given the repetitive nature of these sequences, with traditional pipelines assigning location to thousands of potential genomic regions. This mapping is critical for applicability to the drug development setting to meaningfully treat human disease, and once again picked up on the common theme discussed among the panel of the value and necessity of long read sequencing platforms and approaches.

The conversation precipitated general agreement that ultimately seeking to determine and ascribe functionality and role in human health and disease is critical for application. Efforts should focus on parsing out areas of the dark genome where this can be ascertained, whilst recognising there still may be some of the novel sequence space which may well have little-to-no function.

Moderator: Daniel Rios

*Viral Mimicry – Impact of the Dark Genome on the Innate Immune Response in Autoimmune Diseases, neurodegeneration and cancer*

Opening speaker and panelist: Daniel De Carvalho

Panelists: Andy Satlin

Tomas Mustelin

Dennis Zaller

Andrei Gudkov

Daniel De Carvalho summarized recent findings and highlighted the observation from his groups and many others in the field that disruptions to several cellular processes including DNA methylation, histone modifications, splicing, A-to-I RNA editing, RNA degradation, RNA modifications and RNA-binding proteins can induce an innate immune response named viral mimicry [79, 80]. This response is characterized by a buildup of immunogenic transcripts from endogenous retroelements, which can trigger activation of pattern recognition receptors leading to loss of cell fitness, cell death and a type I/III interferon response that links this innate immune response to a downstream adaptive immune response. Among those endogenous retroelements, Dr. De Carvalho highlighted the role of inverted-repeats alus (IR-alu) pairs. Cryptic transcription of IR-alus due to loss of epigenetic repression or retention of intronic IR-alu pairs due to inhibition of spliceosome leads to formation of dsRNA by intramolecular pairing of the two inverted alus and downstream activation of Melanoma Differentiation- Associated protein 5 (MDA5) - mitochondrial antiviral signaling protein (MAVS) and protein kinase-R (PKR) [81].

Altogether, Dr. De Carvalho proposed the “Fire Alarm Hypothesis”, whereas some endogenous retroelements, such as IR-alus, can be considered triggers for a fire alarm that is activated upon disruptions to core cellular processes. This hypothesis suggests a previously unappreciated tumor suppressor function for endogenous retroelements and suggest that cancer cells with disruptions to these core cellular processes may become dependent in mechanisms to suppress viral mimicry [82, 83]. Dr. De Carvalho showed presented adenosine deaminase RNA specific-1 (ADAR1) as one example of such dependency [81] and suggested that other druggable dependencies may also exist. Finally, he raised the possibility that a range of autoimmune diseases, characterized by over-production of type 1 interferons, could be attributed to non-malignant defects in many of the pathways required for maintenance and sensing of transposable elements leading to misfiring of the viral mimicry response.

A spirited conversation during the panel discussion ensued that touched on two key topics – the relevance of the “fire alarm hypothesis” to cancer, autoimmunity, aging and neurodegeneration, and how these observations can be translated to the clinic.

Several salient points were brought up regarding the “fire alarm hypothesis”. Dr. Mustelin speculated that the at baseline these transposable elements may promote a type 1 interferon “tone” which would enable swift responses in the face of a novel challenge. Dr. De Carvalho noted that several interferon stimulated genes had ALU elements in their 3’UTR which may trigger a feed forward loop for a type 1 interferon response. While Dr. Zaller noted that in the case of autoimmunity we should consider these triggers as “false alarms”, which would suggest that it may be possible to return the system to baseline with little to no unwanted effects.

The topic of clinical translation of Dark Genome research extended from the moderated session into the open question forum. Dr. Satlin (CSO, Transposon Therapeutics) suggested that in the absence of a complete understanding of the mechanistic underpinnings of disease in a preclinical setting, orchestration of precision phase-2 (Ph2) clinical trials would be key to our understanding of disease and the role of transposable elements in disease pathogenesis. Dr. Zaller highlighted the data science component of ROME therapeutics which will enable precise characterization, and quantification of transposable elements as a key aspect of successful clinical translation. This data science driven approach could also allow for “backtranslation” of pre-existing clinical trial data. Drs. Gudkov and Mustlin observed that the theoretical mechanisms of activation of putative therapies might be intrinsically challenging to demonstrate given their indirect effects.

Overall, the panel agreed on the potential for regulation of the Dark Genome to play a role in multiple diseases including cancer, autoimmunity, neurodegeneration and aging. However, it was also apparent that significant hurdles remain to successful translation of this exciting area of research. Events like the Dark Genome Symposium will be critical to increase the profile of a former scientific backwater and facilitate the development of novel therapies across an incredibly wide range of diseases.

Moderator: Sophie Papa

*Dark Antigens in cancer*

Opening speaker and panellist: Kate Chiappinelli

Panellists: Ralf Leonhardt

Robert Manguso

Nina Bhardwaj

Lelia Delamarre

Following on from immunogenicity in disease driven by viral mimicry, Kate Chiappinelli introduced evidence for cancer antigen discovery and modulation for therapeutic development across TEs, specifically focusing on ERVs and cryptic open reading frames ( ORFs). She introduced the evidence that treatment of some cancers with epigenetic modulating drugs DNA methyltransferases inhibitors (DNMTi) and histone deacetylase inhibitors (HDACi) leads to type 1 interferon responses, upregulation of antigen expression machinery, and increased expression of some cancer antigens [84, 85]. Furthermore, treatment of T cells with DNMTi upregulates effector genes, reversing T cell exhaustion and increasing their activity against virally-infected cells and cancer cells [86–88]. Can we take advantage of this increased immunogenicity to target cancer?

We know that some, but not all, cancers demonstrate upregulation of TEs at the RNA level, and level of expression of TEs broadly correlates with antigenicity in samples from The Cancer Genome Atlas (TCGA) [89]. Kate Chiappinelli used examples from ovarian and endometrial cancers to illustrate cancer specific L1 ORF1P and ERV expression [90–92]. Specific examples of these non-canonical antigens include ERV-3 expression driving checkpoint inhibitor efficacy in kidney cancer [74] and ERV-K-Env pulsed dendritic cells (DCs) stimulating ovarian cancer patient T cell activation and cytotoxicity [93].

Indeed, chimeric antigen receptor T cell therapy directed against HERV-K shows activity in breast cancer models, which may translate to direct therapeutic potential [94]. To serve as T cell antigens, peptides derived from translates dark genome ORFs must be processed and presented on human leukocyte antigen (HLA) molecules for recognition by T cells. Evidence in support of this occurring in cancer includes loss of SET Domain Bifurcated Histone Lysine Methyltransferase 1 (SETDB1), an epigenetic regulator of TE expression, resulting in TE upregulation in murine models of lung cancer and melanoma. Resultant HLA class I presentation of peptides and antigenicity was mitigated by knockout of beta-2 microglobulin [95]. Peptide-HLA prediction algorithms demonstrated upregulation of potential HLA-I epitopes in response to DNMTi and HDACi [96]; and CD8 T cell populations in acute myeloid leukemia (AML) patients recognized ERV-derived peptides both pre- and post DNMTi treatment [97]. Lastly, extension of the vast pool of potential antigens beyond TEs has recently been demonstrated through discovery of non-canonical ORFs in cancer [98]. These non-canonical peptides derive from unstable proteins, with evidence that presentation on HLA is up to 5 times more efficient, likely due to instability of the proteins [99].

Finally, the need to build out our limited evidence for differential impact of DNMTi inhibitors on non-canonical epitope presentation in cancer and normal tissue was touched on. The underlying epigenetic instability of cancers as well as compensatory epigenetic repression occurring in repeat regions that show DNA hypomethylation such as vulnerability derived from second level control loss from the SETDB1 pathway makes the likelihood of a wide therapeutic window between cancer and normal tissue high, but further work is needed to bring combination strategies into the clinic.

The panel discussion picked up on many of these themes. For antigens derived from the dark genome on cancer to compete with neoantigens as targets for vaccines or T cell therapies the panel highlighted the need for tumor specificity of peptide expression, homogeneous expression across cancer tissue, and a robust therapeutic window. Particularly for vaccine development, triggering of the innate immune response was considered key.

Next the panel discussed evidence for immunogenicity of TEs. The evidence is robust in murine models with dark genome derived antigenicity of MLV env a key exemplar. Lelia Delamarre stressed that even in murine models these epitopes are variable in their immunogenicity. Evidence in humans is more patchy but exists and is expanding.

The topic of risk and therapeutic windows was then raised, especially in the context of combination therapies, including potential triplets for vaccines, checkpoint inhibitors and DNMTi. Robert Manguso highlighted that the clinical acceptance of off target inflammatory toxicity is relatively high with the adverse events routinely seen with checkpoint inhibitors. Highly cancer specific targets are key to derisking. Ralf Leonhardt emphasised that it is peptide expression that will define normal tissue risk rather than RNA levels. We have limited tools to quantify translation rather than transcription in tissues but newer approaches, such as Ribo-seq are helping enhance our confidence. Nina Bhardwaj made the point that risk is higher with T cell therapies than with vaccines but that we need to broaden and deepen our understanding of normal tissue and rare cell expression of targets of interest to build confidence.

Moderator: Sarah Knutson

*The Dark Genome and gene regulation*

Opening Speaker and panellist: Marie Classon

Panellist: Brad Bernstein

Josh Mandel-Brehm

Danuta Jeziorska

The Dark Genome makes up 98% of our genetic material, and misregulation of these non-coding and repetitive regions Has been implicated in a variety of diseases. We are just scratching the surface in our understanding of Dark Genome regulation: changes in DNA methylation and other epigenetic regulatory mechanisms, the role of transcription factors in tuning the expression of regulatory elements, genomic interactions in 3D and disease -linked variants. How do we illuminate more of the Dark Genome, and apply what we already know to development, disease, and drug discovery? The speakers in this session brought multiple perspectives to key challenges facing the field of Dark Genome regulation.

A key to addressing any disease is to understand if the target of interest is a cause or consequence of disease development. The vastness of the non-coding part of the genome provides an even grander challenge.

Expression of transposable elements and endogenous retroviruses are correlated with cancer and inflammatory diseases. The challenge is determining if and in which context there may be a vulnerability to repetitive element expression, by using genetic tools and small molecule inhibitors (as demonstrated by previous work of the academic, pharma, and biotech panel members). It should also be noted that the diseased cells may have harnessed adaptive mechanisms such as the downregulation of the Stimulator of Interferon Genes (STING) pathway during the evolution of some cancers as an example. Instead of therapeutic targets, we could also consider repetitive element profiles as biomarkers, which would allow for a precision approach for patient selection. As an example, cancer cells that carry an abundance of double stranded Alu RNAs, or other cytoplasmic nucleic acid species (reminiscent of a viral infection), may be more sensitive to the loss of ADAR.

We are also moving toward understanding which mutations in the Dark Genome affect genome function, and the connection to which genes are directly affected in disease. Using this concept, complex disease systems (such as cancer) may not be the first place you start with for drug discovery; instead, starting with monogenic diseases could provide a more streamlined model for proof-of-concept?

Dark Genome elements and peptides are structurally and functionally different between mouse and human, and their regulation is not always conserved between species. This creates a challenge both to our understanding of basic biological mechanisms involving the dark genome and to therapeutic development opportunities that rely on the use of model organisms in this space. Promising efforts have been made to generate mice with large inserts of humanized genomic sequences to mimic the Dark Genome regions in monogenic diseases [100]. While the expression patterns are correct for those regions, the goal would be to achieve disease-level expression through improved cis-elements regulation and even larger genomic regions to correlate to the human disease.

A mouse model of human cancer specifically engineered to upregulate Dark Genome elements through loss of an epigenetic modifier (ref. SETDB1/HERV example) suggests an improved response to checkpoint inhibitors [95]. While the functions of epigenetic regulators may be similar between mouse and human, there is divergent repetitive element structure and function between species, and interpretation of the data would benefit from the compliment of Dark Genome analysis in related human tumor samples.

To delve into the specific regulation of the Dark Genome, the challenge is not necessarily around the existing tools. We have seen an evolution of capabilities to solve these challenges, such as long-read sequencing, single cell gene expression profiling, chromatin structural analysis, and 3D genomic interactions. The technology is available for integration of these “-omics” data sets using predictive modeling and machine learning. It really comes down to sample procurement and data generation. Generating and understanding the data sets required is a significant next step for academia and industry. The ultimate ask is for a Dark Genome database akin to the Encode project. Ideally it would contain bulk and single cell data sets from relevant primary disease models and normal cell systems. And due to the variation of genomes per sample, -seqs from the same sample would allow for an apples-to-apples comparison. By collating databases with standard sets of tools, common annotations, and data sets, we can transform our understanding of Dark Genome regulation, the way these databases transformed the coding genome.

David Ting: *What is the future of the Dark Genome?*

Although we have known about the Dark Genome since the early days of molecular biology, we are just now developing the tools to accurately map these sequences that make up the majority of our genome. For the most part biomedical research has focused on the exome, which is highly conserved between individuals. However, as we develop the next generation of precision biomedicines, the Dark Genome encodes significant genetic heterogeneity that underlies the broad phenotypic variation between individuals. Illuminating the genetic and molecular rules of the Dark Genome will improve patient selection in our use of current drugs and open new therapeutic avenues with refined precision.

Application of current drugs that we know target Dark Genome biology include epigenetic therapies like DNA hypomethylating agents used for cancers and reverse transcriptase inhibitors commonly used for viral therapies. These initial footholds into the druggable landscape of the Dark Genome provide a path to expand refined compounds that can be precisely tuned to target particular parts of repeat element biology. Moreover, the computational tools to accurately quantify the genetic, epigenetic, and transcriptomic patterns of the Dark Genome provide opportunities to better understand the effects of existing therapies and potentially unlock new indications for those assets. Finally, emerging scientific work has revealed the ability of the Dark Genome to infect other cells through extracellular vesicles [101], or resurrection of endogenous retroviruses [102], that could trigger antibody responses to the Dark Genome [18]. The building of these molecular maps and the deeper mechanistic understanding of repeat element biology will open a new dimension to explore the next generation of biomedicines to target the Dark Genome.

The final talk of the meeting was given by Rick Young where he described transcriptional regulatory elements, their protein and RNA components and the multiple points at which they could be leveraged for therapeutic development.

## Conclusion

This inaugural symposium provided an overview of the breadth of human biology impacted by the Dark Genome. It highlighted the importance of technological and platform development to open the door to enhanced understanding. Finally, it touched upon the potential impact on human disease enabled through Dark Genome focused drug development.

## Data Availability

Not applicable.

## Abbreviations

ADAR1: adenosine deaminase RNA specific-1
ALS: amyotrophic lateral sclerosis
*Alu*: Arthrobacter luteus restriction endonuclease sequence
cGAS: cyclic GMP-AMP Synthase
CH3: methyl group
CRE: cyclization recombinase
CSF: cerebrospinal fluid
DCs: Dendritic cells
DNMT1: DNA methyltransferase 1
DNMTi: DNA methyltransferases inhibitors
dsRNA: double stranded ribonucleic acid
*Env*: envelope
ERVs: endogenous retroviruses
GTEx: Genotype- SETDB1Tissue Expression
HDACi: histone deacetylase inhibitors
HERV-K/HML-2: human endogenous retrovirus-K
HERVs: Human endogenous retroviruses
hg38: Homo sapiens (human) genome assembly GRCh38
HLA: human leukocyte antigen
IFN-I: Type I Interferon
LINE (-1): Long Interspersed nuclear element (-1)
LTRs: long terminal repeats
LUAD: lung adenocarcinoma
MAVS: mitochondrial antiviral signaling protein
MDA5: Melanoma Differentiation- Associated protein 5
MLV: murine leukaemia viruses
NRTI: nucleoside reverse transcriptase inhibitor
ORF: open reading frame
ORF1p: LINE-1 open reading frame 1 protein
p53/*Tp53*: Tumor Protein p53
Ph2: phase-2
PKR: Protein Kinase-R
RTE: reterotransposon
SETDB1: SET Domain Bifurcated Histone Lysine Methyltransferase 1
shRNAs: short hairpin RNAs
STING: Stimulator of Interferon Genes
SVA: SINE-VNTR-*Alu*s
TAF1: TATA-box binding protein associated factor 1
*TBXT*: T-Box Transcription Factor T
TCGA: Cancer Genome Atlas
TPRT: target-primed reverse transcription
VLP: virus-like particles
XDP: X-linked dystonia parkinsonism
